# Active control of a powered ankle-foot prosthesis

**DOI:** 10.1186/1757-1146-7-S1-A60

**Published:** 2014-04-08

**Authors:** Ashwin Needham, Andrzej Ordys

**Affiliations:** 1 School of Mechanical and Automotive Engineering, Kingston University, London, UK

## 

Amputees suffer a higher metabolic demand on their bodies. Passive prostheses seek to reduce this deficit through elastic distribution of the energy inherently dissipated in walking. Yet with no capacity to generate torque they lack truly biomimetic function. The active prosthesis is a solution to this and opens up a world of active control in the timing and magnitude of energy return. Being able to also modulate the dynamic character of the replaced joint, in impedance and position, brings us closer to a true model of the ankle-foot complex. And so the ankle-foot complex can be seen to be modelled as a visco-elastic system with loading and unloading phases and active power output.

It was identified that the concept of the active automobile suspension system designed by Bose has the capacity to satisfy these functional demands and so was investigated as a viable model for a prosthetic device. In using this model, the ankle joint was modelled as a motor, driven in drive and dynamo modes to convert an elastic system into an electromagnetic system based around a battery. Low power drain derived from significant power recovery makes this concept particularly interesting. The versatility of this concept is regarded as approaching that of the human beyond those devices that rely on passive or fixed elastic systems. This is active elasticity.

A CAD model was generated along with a corresponding control scheme. The control system is proposed as a viable and innovative concept for future prosthetics with great potential for development.

This proposal makes use of underfoot sensors to determine the displacement of the fore and aft sections of the foot above ground, and is proposed as an effective sensory concept to facilitate handling of various terrain including slopes and stairs. The concept is seen as highly versatile in this respect. Models for both slope and stair walking have been implemented.

An exponential function was proposed as a model for the action of impedance at the ankle. This is a highly versatile function that can be matched, using discrete tuning parameters, to each phase of stance against a majority of samples during level walking. This function also facilitates the smooth and controlled contact between the foot and the ground when combined with the underfoot sensors.

The concept is not fully developed but has been initiated. Much further work is possible.

Thesis Supervisor: Professor Andrzej Ordys

November 2013

**Figure 1:**
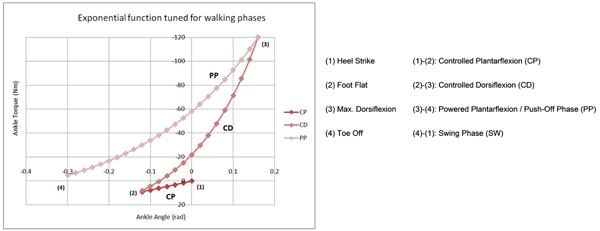
Torque-angle, or stiffness profile derived from exponential function. (Figure 1 inspired largely by [1] as a means of comparison to a typical torque-angle profile.)
